# Microstructural Evolution and Mechanical Response of UHPS Incorporating Steel Slag Fine Aggregate and Different Morphology Steel Fibers

**DOI:** 10.3390/ma19132779

**Published:** 2026-06-30

**Authors:** Jing Wang, Zhiwei Yuan, Yunlong Zhang, Xuesong Qian, Xiaolong Qu

**Affiliations:** 1 School of Transportation Science and Engineering, Jilin Jianzhu University, Changchun 130118, China; 2 Jilin Provincial High Class Highway Construction Bureau, Changchun 130033, China

**Keywords:** shotcrete, UHPC, steel slag, mechanical properties, microstructural characteristics

## Abstract

To mitigate ecological stress caused by natural aggregate extraction and steel slag stockpiling, replacing manufactured sand (MS) with steel slag fine aggregate (SSFA) in ultra-high-performance shotcrete (UHPS) is a promising sustainable strategy. By adjusting the sand–cement ratio (0.7–0.9), steel slag content (0–75%), and the composite ratio of two types of steel fibers. The present study explores the effect of SSFA replacing MS on the mechanical properties of UHPS and conducted a microstructural analysis. While ensuring workability, as the sand–cement ratio increases from 0.7 to 0.9, the mechanical properties of UHPS continue to improve; as the steel slag content increases from 0 to 75%, the compressive strength and flexural strength of UHPS show a gradual upward trend, increasing by 1.15 times and 1.23 times, respectively. The optimal split tensile strength was determined to be at a steel slag dosage of 50%. In comparison with straight steel fibers, hooked-end steel fibers have been shown to enhance compressive strength by an average of 9%, flexural strength by an average of 11%, and splitting tensile strength by an average of 19%. Furthermore, the synergistic effect of steel slag as fine aggregate and steel fibers demonstrated a positive impact on the energy absorption of UHPS. Therefore, an appropriate proportion of steel slag can effectively improve the mechanical properties of UHPS and promote the recycling of industrial solid waste steel slag.

## 1. Introduction

Sprayed concrete technology is recognized as a highly efficient construction technique and has seen widespread application in infrastructure projects, tunnel construction, mining operations, slope stabilization, and various engineering applications. The principal advantages include rapid setting, excellent adhesion, high early strength, and the ability to cover large and complex surfaces [1,2,3,4]. In conventional tunnel engineering, the prevalent use of composite lining structures involves a multi-layer system typically composed of a primary support layer, an intermediate waterproofing membrane, and a secondary lining. Within this framework, the primary support is principally responsible for stabilizing the excavated rock mass; whereas, the secondary lining acts as a safety reserve, bearing long-term loads and preventing seepage. Nevertheless, the conventional composite lining system is subject to significant limitations: insufficient bonding between the primary and secondary linings can hinder stress transfer, thereby reducing the overall support effectiveness. Additionally, the intermediate waterproofing layer often creates water-retaining voids when subjected to complex hydrostatic pressures, which may lead to cracking or leak-age in the lining [5]. Moreover, traditional sprayed concrete tends to develop cracks and experience shrinkage over long-term use, resulting in inferior durability and notable declines in material properties [6]. In contrast, single-layer lining comprises an integrated support structure realized either through a single casting or via successive spraying of high-performance fiber-reinforced shotcrete, such as steel fiber or polypropylene fiber-reinforced concrete. This approach does not simply eliminate the secondary lining but instead embodies an innovative design methodology predicated on advanced high-performance materials, marking a substantial leap towards greater efficiency and reliability in modern tunnel engineering [7].

As the demands of modern tunnel engineering become increasingly sophisticated, single-layer lining structures have evolved, attaining greater prominence and broader adoption. In this context, UHPS has gained substantial research and engineering attention owing to its outstanding mechanical attributes and superior durability. UHPS effectively mitigates the limitations associated with traditional shotcrete in terms of strength and toughness, affirming its potential as an optimal material for permanent support applications within single-layer tunnel linings. Beyond suitability for general support works, UHPS demonstrates exceptional performance in harsh or extreme operational environments, thereby fulfilling more rigorous infrastructure construction requirements [8]. The incorporation of modified constituents including superplasticizers, mineral admixtures, and various fiber reinforcements substantially enhances the fresh workability, mechanical performance, and durability of UHPS [9]. The density and strength of UHPS can be further upgraded through adjustment of the mix proportions and the strategic addition of mineral admixtures, such as silica fume and fly ash [10]. Empirical evidence highlights that the inclusion of steel fibers significantly reduces the rebound rate of sprayed concrete, markedly elevates toughness, and mitigates the risk of cracking [11,12]. The strength of the interfacial bond between steel fibers and the cementitious matrix is a critical determinant of the concrete’s mechanical properties. The fiber pull-out mechanism is contingent upon the fiber’s geometry; notably, hooked-end steel fibers (HFs) exhibit superior interfacial bonding relative to straight steel fibers (SFs), due to the mechanical anchorage afforded by their deformed morphology. Furthermore, optimizing the sand–cement ratio, as well as increasing the fine aggregate content, leads to a denser matrix that substantially elevates the frictional resistance at the fiber–matrix interface, ultimately enhancing the interfacial bond strength [13]. Although UHPS exhibits excellent mechanical performance and durability, its high consumption of natural aggregates and cementitious materials raises concerns regarding resource depletion and environmental sustainability. Therefore, developing sustainable UHPS by incorporating industrial by-products has become an important research direction [14].

The meticulous selection and optimization of aggregates play a pivotal role in the advancement of concrete technology. Employing high-quality crushed stone and natural sand, as coarse and fine aggregates respectively, can substantially elevate the integral performance of concrete [15]. Research has indicated that the use of high-density, high-hardness quartz sand as a fine aggregate not only improves permeability but also augments the compressive strength of concrete [16]. At present, natural sand remains the prevailing fine aggregate in most concrete mixtures. Nonetheless, significant spatial heterogeneity in the distribution of domestic natural sand reserves gives rise to pronounced regional imbalances in supply and demand [17]. The rapid increase in urbanization and large-scale infrastructure development, especially in rapidly developing economies, has driven the growth of international annual natural sand consumption. The global demand for natural sand is immense and unsustainable, with estimates suggesting annual consumption ranging from 40 to 50 billion tons, primarily driven by the construction industry. Based on the cement production of 150 countries in 2017, it can be inferred that the natural sand used for concrete in 2017 alone was between 28.7 and 32.8 billion tons. GAIN estimates global aggregates demand will likely rise to 60 billion tons per annum by 2030 [18]. Current modes of industrial sand extraction precipitate a succession of ecological and environmental challenges: interception of river flow results in wetland degradation, mechanical extraction contributes to substantial dust emissions, and the transport of sand and gravel exacerbates environmental pollution via dust and vehicular exhaust. Particularly in regions such as the Yangtze River Basin and Pearl River Delta in China, excessive sand mining has led to issues such as riverbed deepening and severe riverbank erosion [19]. Against this backdrop, identifying environmentally sustainable alternatives—such as recycled aggregates—has become imperative to future-proofing the concrete industry for sustainable development [20].

Several studies have explored the viability of incorporating industrial by-products, such as steel slag and waste glass, as alternative aggregate sources in concrete [21]. Steel slag, the principal solid by-product of steelmaking, is typically generated in a ratio of approximately 1:3 relative to yearly steel output [22]. Several systematic methodologies exist for its classification; one widely accepted approach categorizes steel slag by its production process, with basic oxygen furnace slag (BOFS) comprising 65–75% of the total, electric arc furnace slag (EAFS) 10–15%, and the remainder attributed to ladle refining slag (LFS) and casting residues [23]. Steel slag production exhibits pronounced geographic variance: as the leading global steel producer, China generated 143 million tons of steel slag (primarily BOFS) in 2022, accounting for roughly half of worldwide output. However, China’s utilization rate remains below 30%, in stark contrast to developed countries like the United States (98%) and Japan (99%) [24]. Consequently, China’s cumulative steel slag stockpile has surpassed 1.2 billion tons, occupying over 36,000 hectares of land and posing considerable land use and environmental hazards [25]. With major components including C2S, C3S, C3A, and Fe_2_O_3_, steel slag possesses latent hydraulic properties [26]. Its physical characteristics, such as a rough, angular surface, high specific surface area, and elevated internal friction angle, significantly enhance the interfacial bond between the cement matrix and aggregate, thereby improving concrete strength. Steel slag can therefore function both as a partial cement substitute and as coarse or fine aggregate in concrete formulations [27]. Experimental investigations have demonstrated that substituting traditional aggregate with steel slag coarse aggregate markedly improves compressive and impact strength [28,29]. Studies by Liu and Guo confirm the beneficial effects of steel slag in UHPC mixtures, reporting increased flowability and superior compressive strength when using steel slag aggregate [30]. Nguyen et al. further corroborate that incorporating steel slag aggregate elevates the compressive strength of concrete and yields satisfactory performance in self-shrinkage and adiabatic temperature rise tests [31]. Although previous studies have confirmed the feasibility of utilizing steel slag in UHPC, most investigations have focused on steel slag powder or independently evaluated steel slag aggregate replacement, while systematic studies on UHPS incorporating steel slag fine aggregate remain limited.

Existing studies have mainly focused on UHPC incorporating steel slag powder or steel slag aggregate, while numerous investigations have also examined the reinforcing mechanisms of steel fibers. However, these factors are generally evaluated independently, and limited attention has been devoted to the preparation and performance optimization of UHPS incorporating SSFA. In particular, the coupled effects of SSFA replacement ratio, sand–cement ratio, and steel fiber morphology on the mechanical properties and microstructural evolution of UHPS have not been systematically investigated.

Therefore, this study aims to prepare sustainable UHPS by replacing manufactured sand with SSFA and systematically evaluating the synergistic effects of SSFA replacement ratio, sand–cement ratio, and steel fiber morphology on compressive strength, splitting tensile strength, flexural behavior, water absorption, and microstructural characteristics. SEM is employed to reveal the strengthening mechanisms associated with the aggregate–matrix interface and fiber–matrix bonding behavior. The results provide theoretical guidance for the high-value utilization of steel slag and the optimized design of sustainable UHPS for tunnel engineering applications.

## 2. Materials and Methods

### 2.1. Materials

**Figure 1:**
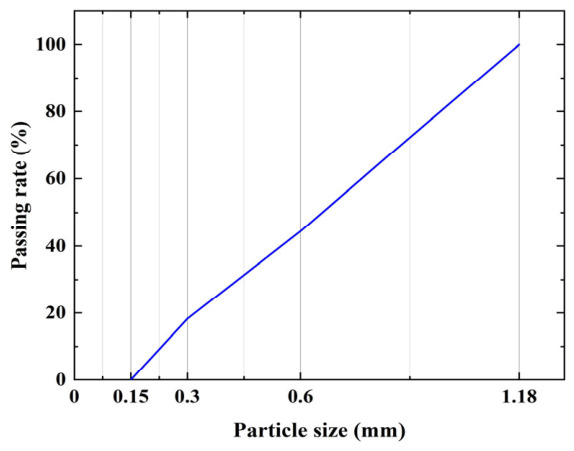
Grading curve of mechanized sand and steel slag aggregate.

**Figure 2:**
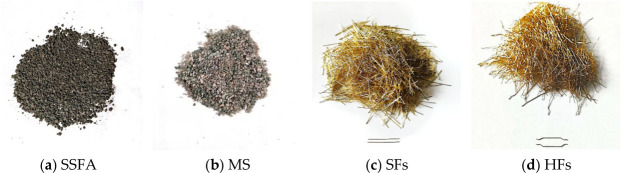
Appearance of the materials.

The constituent materials used for the preparation of UHPS comprised P.O52.5 Portland cement, silica fume, fly ash, metakaolin, manufactured sand, polycarboxylic acid-based superplasticizer, steel slag, steel fibers, and potable water. The composition of cementitious materials is detailed in Table 1. The superplasticizer, a polycarboxylic acid product supplied by Sichuan Dongrun Baisheng New Materials Co., Ltd. (Chengdu, China), displays a water reduction efficacy of no less than 45%. MS and SSFA were used as fine aggregates with a particle size range of 0.15–1.18 mm (Figure 1). The fineness modulus is 2.37, and the specific gravity of MS and SSFA are 2.65 and 3.38, respectively. As shown in Figure 2a,b, SSFA exhibits a rough and porous surface; whereas, MS has relatively smooth and regular particles. Tap water sourced from Changchun City was employed for mixing. The study utilized two types of high-strength copper-plated steel fibers: hooked-end steel fibers (HFs) and straight steel fibers (SFs), both procured from Liaocheng Hongshengyuan Metal Products Co., Ltd. (Liaocheng, China) as displayed in Figure 2c,d. Both fiber types had a diameter of 0.2 mm, a length of 13 mm, a tensile strength of 2950 MPa, and a modulus of elasticity of 200 GPa.

The type of SSFA implemented in this research was basic oxygen furnace slag (BOFS), sourced from Shijiazhuang Xuhan New Material Technology Co., Shijiazhuang, China. The principal chemical composition comprises 40–60% CaO, 10–20% SiO_2_, 20–30% FeO/Fe, 1–6% Al_2_O_3_, and 2–10% MgO, in addition to minor oxides such as MnO, P_2_O_5_, and SO_3_ [32]. Comprehensive chemical constituents are enumerated in Table 2—each proportion lies within established standards. Morphologically, SSFA is predominantly granular and exhibits a water absorption ratio of approximately 3%, which is substantially higher than that of natural aggregates (such as granite: 0.2–1.2%; limestone: 0.3–2%) [27]. Elevated water absorption by SSFA reduces the effective water–cement ratio (w/c), thereby impairing concrete workability; conversely, it promotes a more robust bond between the aggregate and the cement hydration products. Findings by BHJ et al. indicate that a 100% SSFA substitution leads to a slump outside the recommended fluidity range (160–180 mm) [15]. Pavitar et al. reported that a 75% SSFA substitution not only enhanced mechanical properties, but also resulted in a decrease in water absorption compared with natural aggregates [33]. Moreover, Rashad found that replacing natural fine aggregate with 25–100% EAF slag (size 0.063–2 mm) progressively decreased workability. Specifically, mixtures containing 25%, 50%, 75%, and 100% EAF fine aggregate led to slump reductions of 21.61%, 28.71%, 40.32%, and 72.26%, respectively, indicating a clear negative correlation between EAF content and flowability [34]. And in our preliminary experiment, we found that when the substitution rate of steel slag reaches 100%, the fluidity of the mixture decreases significantly, making it impossible to spray. Accordingly, to balance fresh concrete workability and mechanical performance, this study adopted a maximum SSFA substitution rate of 75%. To ensure a valid comparison, all mixtures were proportioned to achieve a target flow value. The free water content was kept constant for all mixes. The varying water demand of the aggregates was compensated for by adjusting the dosage of the superplasticizer, with the steel slag mix requiring a higher dosage to maintain the target workability [35].

### 2.2. Mix Design

To systematically investigate the effects of different design parameters, an orthogonal experimental design was adopted in this study. Three factors, including sand–cement ratio (0.7, 0.8, and 0.9), steel fiber reinforced asphalt concrete (SSFA) substitution rate (0%, 25%, 50%, and 75%), and steel fiber morphology (straight ends and hooked ends), were considered to design a total of 24 different mixing schemes. The orthogonal design enabled the independent evaluation of the influence of each factor and their combined effects on the mechanical performance and microstructural characteristics of UHPS with a relatively limited number of specimens, thereby improving the efficiency and reliability of the experimental program. Table 3 lists the detailed mix parameters. In the symbol representation, S denotes the sand–cement ratio and G signifies the SSFA replacement percentage. For example, S7G25 refers to a sand–cement ratio of 0.7 complemented by a 25% SSFA substitution. In subsequent descriptions, SF-S7G0 indicates a mixture with SFs, a sand–cement ratio of 0.7, and 0% SSFA replacement; HF-S7G0 follows the same convention for HFs.

### 2.3. Specimen Preparation and Test Methods

#### 2.3.1. Specimen Preparation

The specimens investigated in this study were fabricated following the procedures outlined in the Chinese standard JGJ/T 372-2016 [36]. Initially, four types of cementitious binders—cement, silica fume, fly ash, and metakaolin—were introduced into the mixing pot and dry-blended for 2 min to achieve uniformity. Subsequently, a predetermined amount of polycarboxylic acid superplasticizer was dissolved in the designated mixing water, and this solution was then incorporated into the blending pot for an additional 2 min of mixing. The steel fibers were uniformly distributed using the shaking sieve technique, whereby they were sieved evenly into the fresh mix to prevent clumping and to ensure homogeneity throughout the UHPS. The entire mixture was then subjected to four more minutes of mixing to yield the UHPS slurry. The freshly mixed UHPS was immediately transferred to the spraying apparatus for application. During spraying, the nozzle-to-mold distance was maintained at 0.6–1.0 m, with the spray angle set at approximately 80°. The thickness of the UHPS layer at a single spray point was measured as 145 mm, and the rebound rate of concrete at this location was 12.6%. These parameters adhere to the specified requirements for tunnel engineering, attesting to the adequacy and effectiveness of the spraying methodology. After the spraying was completed, subjected to standard curing for 24 h, followed by demolding and outdoor natural curing until the specified testing age (3 d, 7 d, 14 d and 28 d), with ambient temperatures ranging from 16 to 28 °C and an average relative humidity of approximately 65%. The sprayed panels were cut into the required test specimens using a rock cutting machine.

#### 2.3.2. Test Methods

##### Fluidity

**Figure 3:**
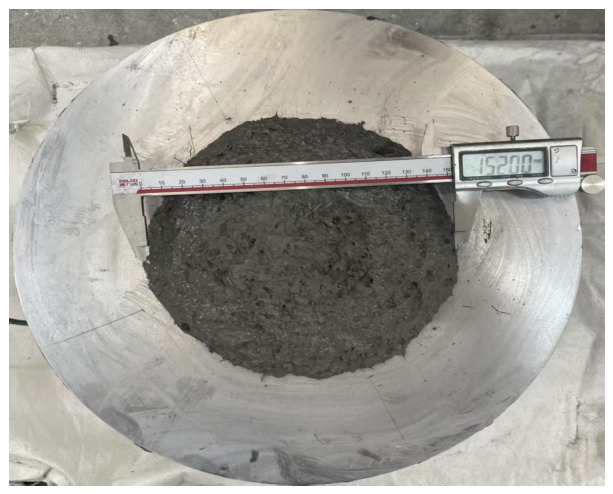
Fluidity test.

Fluidity test was performed according to the “Method for Determining the Fluidity of Cement Mortar” (GB/T 2419-2005) [37], utilizing a mortar flow table apparatus, commonly referred to as a jumping table. Upon completion of 25 standard-frequency impacts on the flow table, the diameters of the dispersed mortar in two orthogonal directions were measured utilizing a digital vernier caliper, and the arithmetic mean was recorded as the final flow value. The experimental procedure is exemplified in Figure 3. The results indicate that the flow values of cement mortar across varying mix proportions remained consistently within the range of 14–18 cm, which meets the operational requirements for shotcrete applications according to previous conclusion [38].

##### Mechanical Properties

**Figure 4:**
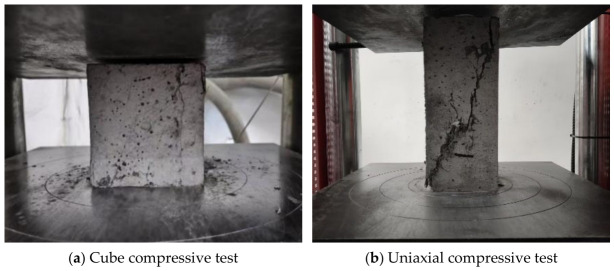

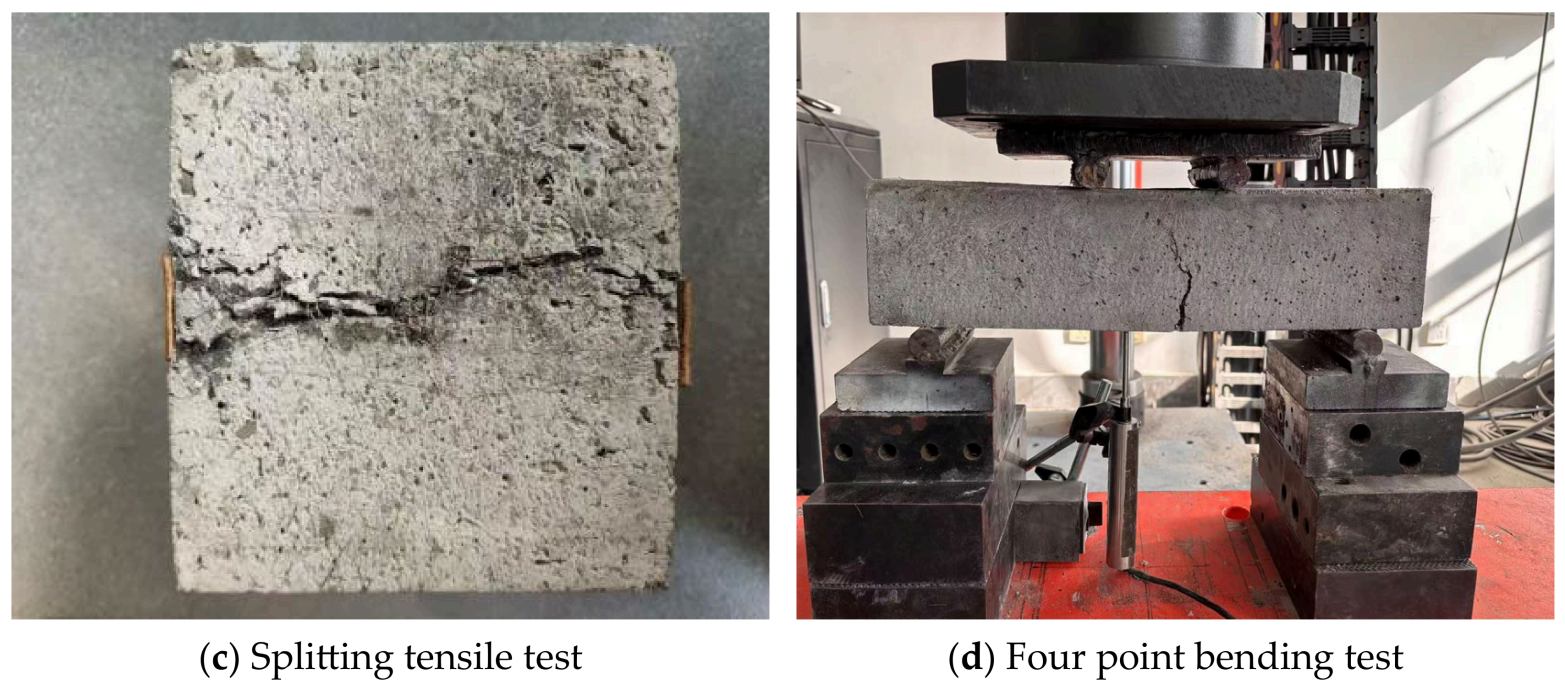
Mechanical property test.

Upon reaching the designated test age, mechanical testing was executed in accordance with Chinese standards JGJ/T 372-2016 [36] and GB/T 50081-2019 [39]. For compressive and splitting tensile strength assessments, cube specimens (100 mm × 100 mm × 100 mm) were prepared, with three replicates per experimental group. The loading rates applied during cube compressive strength and splitting tensile strength tests were 1.0 MPa/s and 0.1 MPa/s, respectively. Uniaxial compressive strength was measured using prismatic specimens of dimensions 100 mm × 100 mm × 300 mm, also in triplicate per group. Flexural performance was evaluated using prismatic specimens measuring 100 mm × 100 mm × 400 mm with a testing span of 300 mm. The rate of the four-point bending test is 0.1 mm/min. Portions of the test process are illustrated in Figure 4.

##### Water Absorption Test

Water absorption evaluation was conducted following Chinese standard GB/T 50081-2019 [39], commencing at the conclusion of a 28-day curing period. The assessment used standard cube specimens (100 mm × 100 mm × 100 mm; 3 specimens per group). Samples were submerged in a thermostatic water bath maintained at 20 °C for 24 h. After soaking, surface moisture on specimens was removed and weights were recorded. This process was repeated until the mass change between two consecutive 24 h intervals was less than 0.2%, with a minimum soaking time requirement of 48 h. Subsequently, specimens were dried in an oven at 105 ± 5 °C for 24 h, again recording weights at 24 h intervals until mass variation remained under 0.2%. The final water absorption ratio for each specimen was then calculated.

## 3. Results and Discussion

### 3.1. Cube Compressive Strength

**Figure 5:**
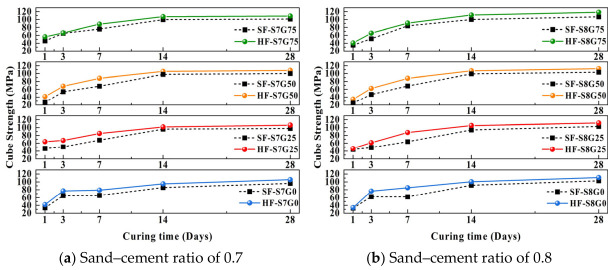

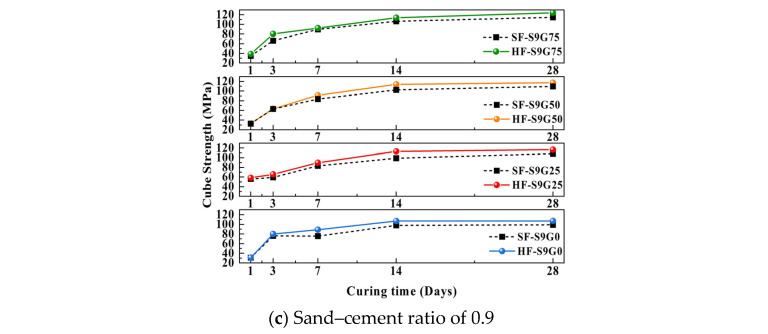
Cube compressive strength at 1 d, 3 d, 7 d, 14 d, and 28 d.

The cube compressive strength (f_cu_) of UHPS investigated in this study was determined using cube samples with dimensions of 100 mm × 100 mm × 100 mm. The results depicted in Figure 5 indicate that the influence of steel fiber type, sand–cement ratio, and the replacement rate of steel slag fine aggregate (SSFA) with mechanical sand (MS) at various ages were comprehensively evaluated, and the results were systematically plotted. As shown in Figure 5, the highest compressive strength in the test reached 124.08 MPa, which meets the minimum strength requirement for UHPC in Chinese standard T/CECS 864-2021 [40].

#### 3.1.1. The Effect of Curing Time on the Cube Compressive Strength

As illustrated in Figure 5, all UHPS specimens within the same group exhibited continuous increases in compressive strength with extended curing time, peaking at 28 days. This trend primarily occurs because prolonged curing facilitates the progressive hydration of all cementitious constituents. The remaining unhydrated C_2_S undergoes further reactions, producing C-S-H gel that accumulates and intertwines into a dense network, effectively filling pores and strengthening interfacial bonding. Additional crystallization of calcium hydroxide (CH) and ettringite (AFt) further enhances the density of the matrix [41,42]. Furthermore, the presence of fly ash can encapsulate cement particles, temporarily isolating them from water; as water gradually permeates and reacts with these encapsulated particles, compressive strength is further promoted. By comparing compressive strength variations among different groups at multiple curing ages, it is evident that the compressive strength of UHPS increases more rapidly prior to 14 days. This can be attributed to the use of silica fume and metakaolin—both rich in SiO_2_ and Al_2_O_3_—in the cementitious system, which accelerate the hydration of C3A and C3S and foster the formation of C-A-H and C-S-H gels, significantly enhancing early strength [43,44,45]. After 14 days, the rate of strength gain tends to stabilize, indicating an approach to hydration equilibrium in the UHPS. Notably, as presented in Figure 5, the early-age (3-day) compressive strength of all test specimens exceeded 46 MPa, and by 28 days, the highest recorded compressive strength surpassed 120 MPa. In comparison with conventional shotcrete, UHPS demonstrates marked superiority in both early and stabilized compressive strengths. This enhanced performance is largely attributable to the elimination of coarse aggregates during preparation, which leads to a denser matrix microstructure, consistent with the closest packing theory underpinning UHPS formulations.

#### 3.1.2. Effect of SSFA Content on Cube Compressive Strength

Analysis of Figure 5, focusing solely on the effect of SSFA replacement rate, reveals that at 1 and 3 days of curing, groups with SSFA replacement rates of 25% and 0% exhibited higher compressive strengths, respectively. However, by 7 days and beyond, the group with 75% SSFA replacement exhibited superior compressive strength compared to other groups, corroborating results reported by BHJ et al. [15]. This demonstrates that SSFA, when utilized as a partial substitution for MS in the preparation of UHPS, confers advantages in both early age strength and later stage strength development.

**Figure 6:**
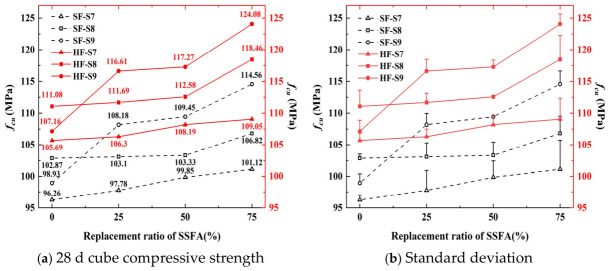
28 d cube compressive strength and standard deviation.

Figure 6a presents the influence of SSFA replacement levels (0%, 25%, 50%, 75%) on the 28-day compressive strength of UHPS, while Figure 6b provides the corresponding standard deviations of compressive strength results. The 50% SSFA replacement level actually exhibits a smaller standard deviation compared to other replacement ratios, indicating reduced variability in compressive strength. This indicates that the material properties are more uniform and reliable at this time. The enhanced consistency may be due to the optimized particle packing density achieved by mixing two types of fine aggregates in equal proportions, reducing local variations in hydration and resulting in a more uniform microstructure. It is apparent from Figure 6 that increasing the proportion of SSFA as a replacement for MS yields a corresponding rise in UHPS compressive strength. Specifically, for the experimental group with a sand–cement ratio of 0.9, incorporation of 25% SSFA resulted in a 9% increase in f_cu_; whereas, a 75% substitution led to the maximum compressive strength increment, marginally surpassing 16% (an absolute increase of 16.9 MPa) relative to the control group. The improvement effect plateaus for SSFA contents between 25% and 50%. This enhancement is largely attributable to the higher density and specific gravity of SSFA compared to MS, with concrete incorporating SSFA exhibiting bulk densities from 2400 to 2520 kg/m^3^, in contrast to the 2340 kg/m^3^ typical for MS-only mixes [46]. As corroborated by prior studies, aggregates of higher specific gravity are more suitable for producing high-strength concrete [24]. Additionally, SSFA differs morphologically from natural aggregates, possessing rough, porous surfaces that yield higher internal friction angles and larger specific surface areas. This contributes to stronger interfacial bonding with the cementitious matrix and improves the mechanical properties of UHPS [47,48,49].

#### 3.1.3. Effect of Fiber Shape on Cube Compressive Strength

The incorporation of steel fibers plays a significant role in determining the compressive strength of UHPS. Steel fibers act as bridges across microcracks, redirecting crack propagation paths and thus impeding the development of damage. Nonetheless, excessive steel fiber content can create additional entrapped air and lead to fiber agglomeration, diminishing strength benefits and potentially impacting both the workability and matrix strength of the UHPS [50]. Balancing material cost and workability, the steel fiber content was fixed at 2% in this study. Analysis of Figure 5 demonstrates that, regardless of the steel fiber type introduced, the overall trend in compressive strength remained consistent. However, comparing groups with identical sand–cement ratios but different fiber shapes revealed that concrete enhanced with 2% hooked-end steel fibers (HFs) consistently outperformed that incorporating straight steel fibers (SFs). Our observations align with those of Weina, Zhang, and others [50,51], who assert that deformed fibers, such as HFs, exhibit superior pull-out resistance and thus provide more effective crack-bridging under loading. As further validated in Figure 6, all other factors being equal, UHPS containing 2% HFs achieved compressive strengths as much as 9% higher than mixtures containing 2% SF, which concurs with results reported by Ying et al. [52]. Prior research has established that adding 2.0% HF (aspect ratio 65) can elevate compressive strength by 8–12%, whereas the use of SFs yields a benefit of only 3–5%. This disparity derives from the propensity for straight fibers to slip or be pulled out at early loading stages, relying solely on interface friction and chemical bonding. In contrast, HF’s hook-shaped ends generate a mechanical anchoring effect that produces robust mechanical interlocking within the concrete. This contributes to enhanced load transfer and a pronounced delay in crack propagation under compression [53].

#### 3.1.4. Effect of Sand–Cement Ratio on Cube Compressive Strength

The impact of varying sand–cement ratio on the compressive strength of UHPS is clearly illustrated in Figure 6. For constant conditions except for sand–cement ratio variations, it is observed that (except for groups without SSFA, which achieve maximum compressive strength at a sand–cement ratio of 0.8) groups containing SSFA demonstrate a consistent trend: compressive strength increases progressively as the sand–cement ratio rises from 0.7 to 0.9. Optimization of this ratio to 0.9, coupled with optimal SSFA content, results in a notable enhancement, with SFs and HFs reinforced UHPS increasing compressive strength by 13.3% and 13.8%, respectively, compared to their performance at a ratio of 0.7. This improvement is attributed to the improved gradation between cementitious and aggregate phases, which reduces voids and enhances density. In addition, finer sand particles provide a filling effect that restrains the formation and extension of microcracks [54].

In summary, the interaction among sand–cement ratio, steel fiber morphology, and SSFA content exerts a significant synergistic influence on the compressive strength of UHPS. An increased sand–cement ratio optimizes the gradation and packing density between fine aggregates and cementitious materials; HF strengthens fiber–matrix bonding via mechanical anchorage, substantially mitigating crack development; and a 75% SSFA replacement rate further densifies the microstructure by reducing porosity in the interface transition zone. Under these mutually reinforcing effects, compressive strength peaks at 124.08 MPa—a 29% increase relative to the reference group—demonstrating a collaborative compaction, reinforcement, and crack optimization mechanism.

### 3.2. Uniaxial Compressive Strength

**Figure 7:**
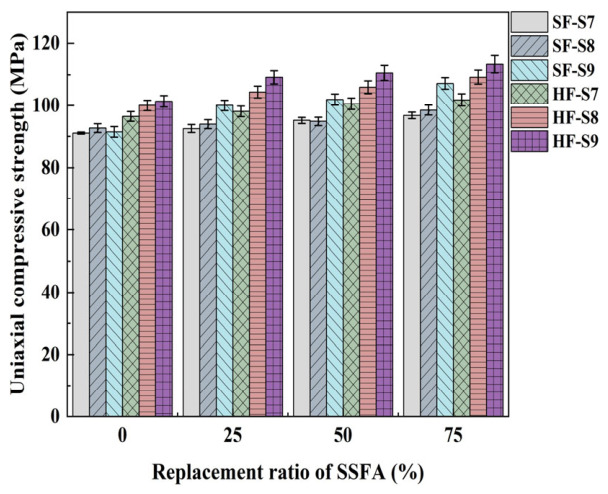
Uniaxial compressive strength.

Uniaxial compressive strength, defined as the maximum sustained compressive stress under pure axial loading, is a fundamental parameter for evaluating material load-bearing capacity and the safety of structural elements under axial forces. The prismatic specimens evaluated here measured 100 mm × 100 mm × 300 mm. Figure 7 presents uniaxial compressive strengths for various mix designs. Results indicate that the uniaxial compressive strength of UHPS follows similar variation patterns to the cube compressive strength. When combining an optimal sand–cement ratio, hooked-end steel fibers (HFs), and appropriate SSFA content, the H-S9G75 group exhibited the highest uniaxial compressive strength (113.41 MPa); whereas, the S-S7G0 group recorded the lowest (91.03 MPa). Groups with higher SSFA content generally achieved a 6–17% gain in uniaxial compressive strength under comparable conditions. Similarly, HF-reinforced groups consistently outperformed SF-reinforced analogs by an average of 8.2%, reflecting the same trend observed for cube compressive strength. The enhancement is primarily due to the enhanced anchoring and interfacial bonding generated by the hooked ends of HFs, which outperform the smooth ends of SFs. Notably, prior studies have demonstrated that HF-matrix bond strength may reach approximately seven times that of SF [55].

**Figure 8:**
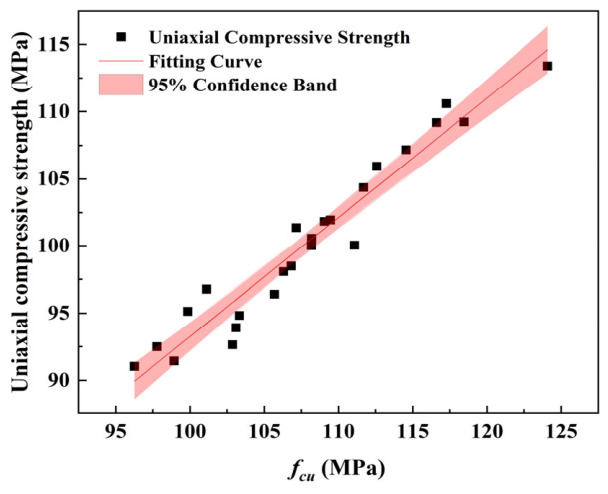
Relationship between cube compressive strength and uniaxial compressive strength.

Figure 8 illustrates the correlation and fitted regression curve between cube compressive strength and uniaxial compressive strength for UHPS mixtures. Given the greater complexity associated with uniaxial compression testing, establishing a reliable relationship between these two measures in SSFA-containing UHPS is essential for practical engineering evaluations. Linear regression analysis revealed a strong correlation between these two parameters, such that y = kx, as described by the regression formula below:

(1) $$f_{c}=0.887f_{cu},R^{2}=0.938$$

In the formula $f_{c}$ is the uniaxial compressive strength, $f_{cu}$ is the cube compressive strength, $R^{2}$ represents the model fitting effect analysis.

The results indicate that for every 1 MPa increase in the compressive strength of UHPS cubes containing SSFA, the uniaxial compressive strength increases by approximately 0.887 MPa. Previous studies have statistically determined that the conversion coefficient between the uniaxial compressive strength and the cube compressive strength of high-strength concrete is approximately 0.9 [53], which is consistent with the conclusions drawn from this experiment. The proposed regression model was established based on the experimental results of this study and is applicable to UHPS mixtures with sand–cement ratios of 0.7–0.9, SSFA replacement ratios of 0–75%, and a steel fiber volume fraction of 2%. As an empirical relationship derived from a limited dataset, its applicability beyond these ranges has not been validated. Therefore, the proposed equation should be regarded as a preliminary predictive model and its applicability beyond the investigated parameter range requires further validation.

### 3.3. Splitting Tensile Strength

**Figure 9:**
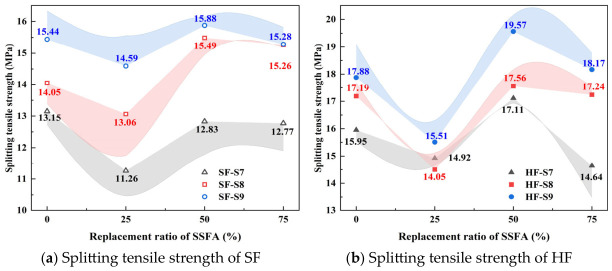
Splitting tensile strength.

Splitting tensile strength at 28 days was analyzed for UHPS specimens, with results depicted in Figure 9. The incorporation of hooked-end steel fibers (HFs) was especially effective in enhancing splitting tensile strength. When isolating the impact of sand–cement ratio, results demonstrate that increasing this ratio from 0.7 to 0.9 significantly enhances the splitting tensile strength, with the HF-reinforced group at 0.9 exhibiting much higher values than corresponding controls, as shown by the pronounced curve in Figure 9.

Examining the role of SSFA replacement rate on splitting tensile strength was one of the focuses of this experimental study. Figure 9 demonstrates a nonlinear relationship as SSFA replacement increased from 0% to 75%. Specifically, the splitting tensile strength of each UHPS group exhibited an initial decline, followed by an increase and then either plateauing or decreasing again, a pattern inconsistent with simple positive correlation but corroborated by previous results [15,56]. Notably, UHPS with 25% SSFA typically showed diminished splitting tensile strength—an average reduction of 1.5 MPa, up to 2.7 MPa, corresponding to 6–16% loss. In contrast, a 50% SSFA replacement improved performance by up to 1.7 MPa (about 3–10% over control). At 75% SSFA, strength was maintained at or exceeded control group levels, aligning with the conclusions of Pavitar et al. [33]. Compared with the reference mixture, the splitting tensile strength exhibited a slight reduction at an SSFA replacement ratio of 25%. A possible explanation is that the aggregate skeleton and interfacial transition zone (ITZ) were not fully optimized at this initial replacement level. At this replacement level, the angular SSFA particles are insufficient to form a continuous interlocking structure, while the coexistence of manufactured sand and SSFA increases the heterogeneity of the aggregate distribution. These factors may have promoted localized stress concentration and microcrack initiation under tensile loading, thereby contributing to the observed reduction in splitting tensile strength. At a 50% replacement ratio, the combined use of SSFA and manufactured sand may have promoted a more favorable particle packing condition and a more uniform matrix structure. This highly homogenized structure maximally resisted crack initiation, overcoming the negative effects of weak interfaces and resulting in peak strength. Comparatively, SSFA’s positive effect is more pronounced for compressive strength than for splitting tensile strength. The statistical analysis shows that the splitting tensile strength results exhibit small standard deviations, all below 1.5, indicating good repeatability and reliability of the experimental measurements. The standard deviation further confirms the validity of the test results.

### 3.4. Flexural Strength

#### 3.4.1. Flexural Load–Displacement Curve

**Figure 10:**
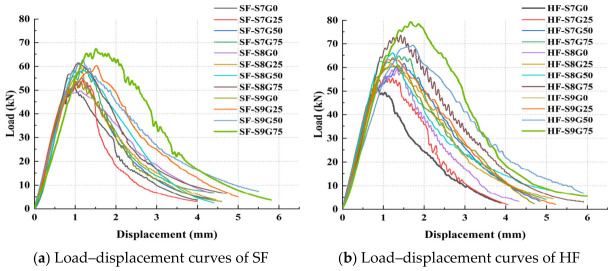
Flexural load–displacement curve diagram.

The load–displacement responses of UHPS incorporating either SFs or HFs at incremental sand–cement ratios (0.7 to 0.9) and varying SSFA contents are displayed in Figure 10. The flexural response of UHPS in four-point bending can be summarized in four distinct stages [53]: Initially, the load–displacement curve is concave, attributable to incomplete contact between the machine and the substantial cross-section of the specimen, as well as the presence of inherent microcracks and initial elastic deformation. In the second stage, the curve rises linearly, representing the elastic regime where the steel fibers and UHPS matrix form an effective synergistic system, thus maximizing composite stiffness. In the third stage, the slope decreases and microcracks propagate, eventually concentrating into a main crack (width 2–3 mm) at midspan. The dominant crack-bridging role of steel fibers initiates, and secondary peaks may appear due to their effective reinforcing action, allowing specimens to carry additional load after initial cracking. In the fourth, post-peak phase, the curve exhibits a sharp followed by a gradual descent; primary and vertical cracks propagate, and the persistent dissipation of energy by fiber pull-out imparts residual bearing capacity [57].

#### 3.4.2. Effect of SSFA Content on the Flexural Strength of UHPS

**Figure 11:**
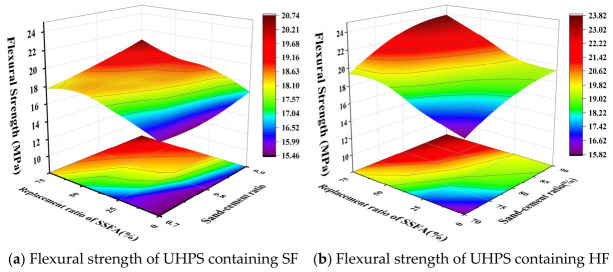
Flexural strength.

Figure 11 summarizes the changes in UHPS flexural strength during four-point bending tests. The analysis demonstrates that increasing SSFA content effectively enhances flexural strength for specimens with identical steel fiber content. This observation is in agreement with conclusions drawn by Maslehuddin [46]. Visualization of Figure 11 reveals that test groups with both high sand–cement ratios and high SSFA replacement rates display more saturated coloration, indicating higher flexural strengths; whereas, lower ratios correspond to lighter hues and diminished strength. Notably, specimens with higher SSFA showed, on average, a 3.6 MPa increase in flexural strength versus low-SSFA samples. This improvement is attributed to the superior adhesion of SSFA aggregates to the cement matrix. SSFA’s irregular surface morphology confers a large specific surface area, enhancing mechanical interlocking and bond strength in contrast to MS, and its hardness further elevates flexural performance [58]. Consequently, it can be concluded that high SSFA content has a pronounced and beneficial effect on UHPS flexural strength, with the maximum strength value recorded at 23.81 MPa for 75% SSFA.

**Figure 12:**
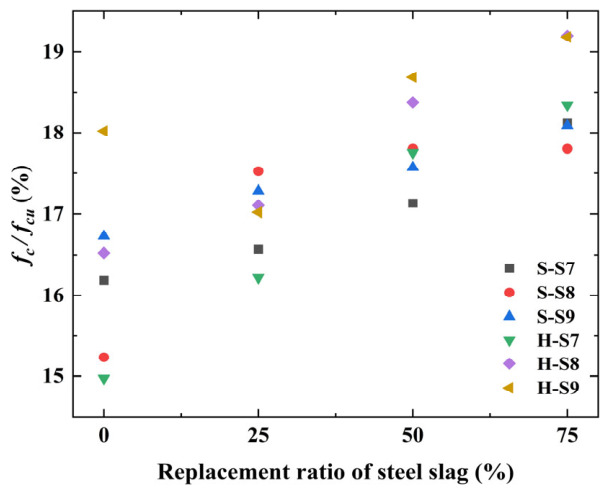
Flexural-compressive strength ratio.

The flexural compressive strength ratio (*f_c_*/*f_cu_*) is an important parameter for evaluating the comprehensive mechanical properties of concrete materials. It reflects the ductile or brittle characteristics exhibited by concrete when subjected to bending loads. A higher flexural strength ratio indicates that concrete has better flexural performance and is less likely to undergo brittle fracture under bending loads. Figure 12 shows the relationship between the flexural strength ratio and the SSFA ratio in the test. For the flexural strength ratio, adding more SSFA can improve the flexural strength ratio. The results demonstrate that SSFA positively affects the flexural strength of UHPS.

#### 3.4.3. Effect of Fiber Shape on the Flexural Strength of UHPS

Table 4 provides a comparative analysis of steel fiber morphologies and their effect on the flexural behavior of UHPS. Taking the S9G75 test group (peak flexural strength at 20.72 MPa) as a reference, the HF modified group achieved a peak flexural strength of 23.81 MPa—a 15% improvement. This comparison indicates that HF provides a more pronounced strengthening effect on flexural properties than SF. This is primarily attributable to the higher pull-out resistance of HF, which restrains interface slippage and facilitates robust crack bridging. However, examination of initial crack strength and deflection reveals that, with constant fiber content and aspect ratio, the influence of fiber geometry on initial cracking is negligible. Studies by Wu, Yoo, and colleagues corroborate this, indicating that initial crack strength predominantly depends on the matrix properties, while steel fibers become the principal factor only after cracking ensues [59,60].

#### 3.4.4. Effect of Sand–Cement Ratio on the Flexural Strength of UHPS

**Figure 13:**
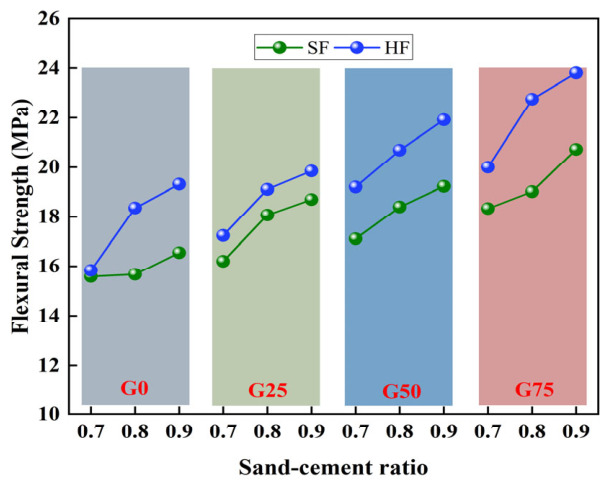
Relationship between sand–cement ratio and flexural strength.

As depicted in Figure 13, the flexural strength of UHPS is greatly influenced by variations in the sand–cement ratio. The effect parallels the trends observed for compressive strength: increasing the sand–cement ratio from 0.7 to 0.9 results in notable flexural strength gains. When other variables are held constant and the ratio reaches 0.9, flexural strength rises by 2–4 MPa compared to the lowest group, amounting to a nearly 20% increase. This performance can be attributed to the exclusive use of fine aggregates in this study, which, when used at higher sand–cement ratios, generate denser packing and a more compact matrix microstructure. Furthermore, improved mortar coverage of sand particles strengthens the interface transition zone. Enhanced sand–cement ratios have also been shown to boost fluidity, facilitating bubble release during molding and thus further increasing density and bond strength. Consequently, at a sand–cement ratio of 0.9, the synergistic effects of steel fibers and SSFA optimally augment UHPS flexural performance.

#### 3.4.5. Evaluation of Bending Toughness of UHPS Containing SSFA

The flexural toughness index was calculated in accordance with CECS13:2009 (Standard Test Methods for Fiber Concrete) [61], with results summarized in Table 5—indices I5 and I10 quantify flexural toughness. The data indicate that both fiber geometry and SSFA content significantly influence these toughness indices, with fiber shape exerting a more prominent effect than aggregate replacement. In comparison to SF, HF consistently outperforms, with toughness indices increasing by up to 14.2% (I5) and 15% (I10). As described by Weina et al. [50], specimens reinforced with HFs can sustain higher loads post-crack initiation; after matrix cracking, stress at the interface is transferred primarily to bridging steel fibers. The crack-bridging behavior is characterized by progressive debonding and fiber pull-out, and HF provides superior mechanical anchorage and pull-out resistance—attributes that greatly enhance overall specimen toughness. These characteristics contribute to improved crack control and enhanced long-term durability of tunnel linings. In addition, it provides higher energy absorption capacity and damage tolerance, providing greater safety margin to cope with dynamic loads and unexpected impacts. Therefore, this increment represents an improvement in the actual performance of tunnel lining.

### 3.5. Water Absorption

**Figure 14:**
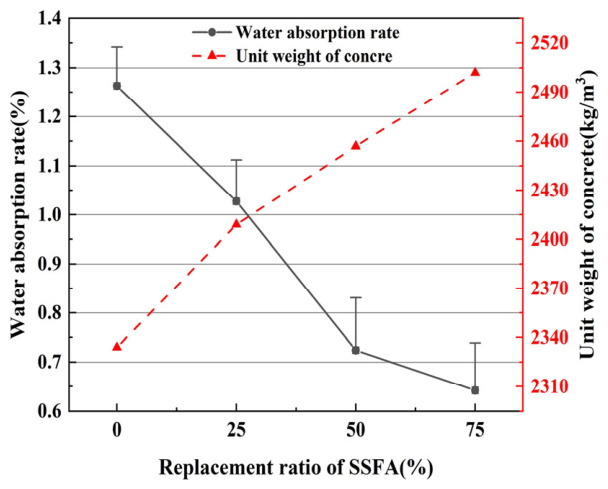
Relationship between SSFA replacement rate and water absorption.

Figure 14 illustrates the influence of SSFA substitution on unit weight and water absorption for HF-reinforced UHPS, with the sand–cement ratio optimized at 0.9. The results clearly demonstrate an increase in unit weight as steel slag content rises from 0% to 75%, while water absorption declines from 1.2% to 0.7%. The water absorption test serves as an indirect indicator of pore structure and capillary absorption characteristics. This trend suggests that the introduction of SSFA not only fills matrix pores, thereby contributing to higher unit weight, but also reduces the number of detrimental pores and strengthens aggregate–hydrate bonding, which collectively promote a more compact matrix microstructure and consequently improve overall mechanical performance. The statistical analysis indicates that standard deviations of the water absorption results for each group are around 0.1, suggesting that the data dispersion is relatively limited and the experimental measurements exhibit good reproducibility. However, since water absorption testing was conducted only for the optimal mixture series, these observations should be interpreted within the scope of the investigated mixtures.

### 3.6. Microstructural Characteristics

The macroscopic mechanical performance of UHPS is closely related to its microstructural characteristics, including aggregate morphology, interfacial transition zone (ITZ) and fiber–matrix bonding behavior. Experimental results showed that increasing SSFA content significantly enhanced compressive strength, while HFs remarkably improved splitting tensile strength, flexural strength, and toughness. To reveal the underlying strengthening mechanisms, scanning electron microscopy (SEM) was employed to investigate the microstructural evolution of UHPS incorporating 75% SSFA and 2% steel fibers.

The SEM observations indicate that the combined use of SSFA and steel fibers optimizes the internal structure of UHPS through multiple mechanisms. The irregular surface structure and porous properties of SSFA provide additional sites for the deposition of hydration products, which may contribute to the formation of denser matrices and more compact ITZ. Simultaneously, the bridging effect of steel fibers effectively restrains crack initiation and propagation, while hooked-end fibers further enhance load transfer through mechanical anchorage. These synergistic effects refine the pore structure, improve interface bonding.

#### 3.6.1. Fine Aggregate

**Figure 15:**
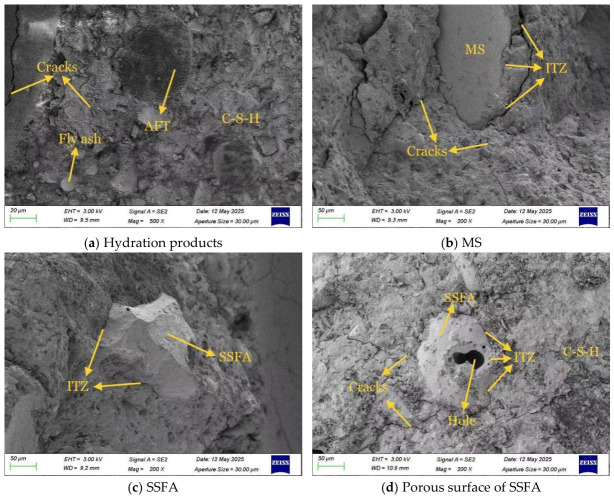
Microscopic images of MS and SSFA.

The primary fine aggregates utilized in this investigation comprised MS and SSFA. As indicated in Figure 15, post-testing microscopic differences are evident. Figure 15a reveals clearly discernible hydration products—C-S-H and AFt—formed by fly ash and other cementitious components, which act as pore fillers and binders between MS and SSFA, yielding a denser structure. Figure 15b presents the microstructure of MS; whereas, Figure 15c,d portrays SSFA within the cement matrix. For each mixture, multiple representative SEM images were selected and the ITZ thickness was measured at multiple locations at the aggregate–matrix interface using Image-Pro Premier 9.2. A total of 30 measurements were taken for each mixture. SEM analysis shows that MS in UHPS exhibits a relatively smooth surface and a more regular shape, while SSFA exhibits irregular, rough, and polyhedral features (Figure 15c), as well as a highly porous surface (the maximum pore size is equivalent to a circle with a diameter of 39 um), and hydration products are distributed in these pores. The average ITZ thickness of SSFA is about 15 um, which is significantly narrower than the average 20 um of MS.

SEM observations further reveal that the rough and angular surface of SSFA creates a stronger mechanical interlocking effect than MS. Meanwhile, the porous surface of SSFA accommodates hydration products such as C-S-H gel and AFt, which effectively fill micro voids and strengthen the aggregate–matrix interface. As a result, the ITZ becomes denser and more continuous, reducing weak zones within the composite. These microstructural improvements explain why UHPS containing higher SSFA contents exhibits significantly enhanced compressive strength and lower water absorption. The reduction in pore connectivity also contributes to the improved compactness of the matrix, thereby providing a more favorable environment for stress transfer under external loading.

#### 3.6.2. Steel Fiber

**Figure 16:**
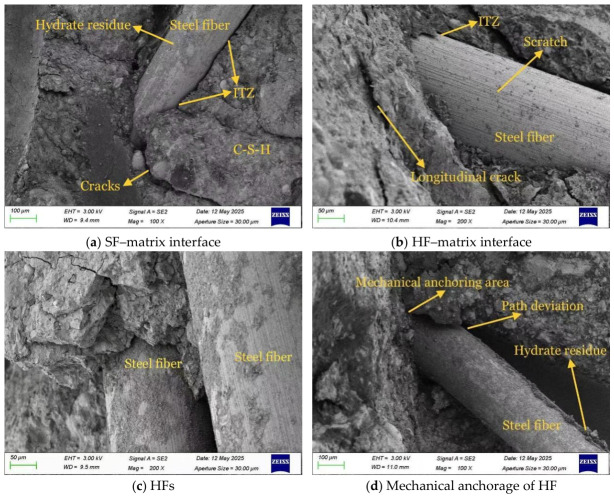
Micrograph images of steel fibers.

Significant mechanistic differences exist between SFs and HFs in their influence on the mechanical performance of concrete. As illustrated in Figure 16 (Figure 16a: SF; Figure 16b–d: HF), both types of steel fibers display vertical surface striations formed during initial loading and crack formation—indicative of interface slippage between fibers and the cement matrix. Such interaction allows the fibers to bridge evolving cracks and contribute fundamentally to strength improvement. The presence of hydrated product residues on fiber surfaces, observed in Figure 16, further affirms strong bonding with the matrix. Notably, comparison of interfacial regions for the two fiber geometries reveals no significant differences in ITZ quality. Furthermore, longitudinal cracking was more frequently seen near the fiber–matrix interface, likely arising from stress concentrations due to the elastic modulus mismatch between fibers and the surrounding matrix, thus initiating microcracks. As depicted in Figure 16c,d, HFs accomplish enhanced reinforcement via both self-integration and mechanical anchoring with the matrix, in contrast to SFs.

**Figure 17:**
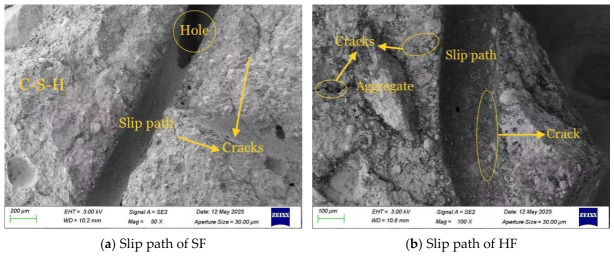
Microscopic images of fiber slip path.

As illustrated in Figure 17a, which depicts the channel left after SF pulling, there are observable holes on the slip path. This phenomenon can be attributed to the obstruction of steel fibers by aggregate during the pulling process, resulting in the displacement of aggregate beneath the load’s influence. The slip path depicted in Figure 17b features a turning angle that corresponds to the hook-shaped end of the HF. This feature enables an augmented contact area between the cement matrix and the HFs, thereby engendering an effective mechanical anchoring effect. As demonstrated in Figure 17, under load, the fiber’s slippage leads to the surrounding matrix being subjected to shear stress, resulting in the formation of multiple radial cracks along the slip path. This phenomenon is attributed to the combined effects of interface friction and mechanical interlocking.

SEM image analysis demonstrates that the incorporation of SSFA and steel fibers produces a pronounced synergistic strengthening effect on UHPS. SSFA primarily enhances compressive strength by densifying the matrix and improving the aggregate–matrix interface. The reduction in ITZ defects effectively limits stress concentration and delays microcrack initiation. Steel fibers mainly improve tensile and flexural performance through crack bridging and energy dissipation mechanisms. During loading, fibers transfer stress across developing cracks, thereby suppressing crack opening and slowing crack propagation. Compared with straight fibers (SFs), hooked-end fibers (HFs) provide additional mechanical anchorage owing to their end geometry, resulting in higher pull-out resistance and stronger fiber–matrix interaction. The SEM images of fiber pull-out paths clearly show that HFs induce multiple radial microcracks and greater matrix engagement, allowing more energy to be consumed during pull-out.

Furthermore, the synergistic interaction between SSFA and HFs produces a more uniform stress distribution throughout the matrix. The dense aggregate–matrix interface established by SSFA provides a stable load-transfer path, while HF effectively bridges and arrests crack propagation. Consequently, UHPS containing 75% SSFA and hooked-end steel fibers exhibits superior compressive strength, splitting tensile strength, flexural strength, and toughness, which is in good agreement with the macroscopic experimental results.

## 4. Conclusions

This study systematically investigated the preparation and mechanical performance of UHPS incorporating steel slag fine aggregate (SSFA). The effects of varying SSFA content—specifically 0%, 25%, 50%, and 75% substitutions—on the overall performance of UHPS were thoroughly examined under different sand–cement ratios (ranging from 0.7 to 0.9) and with the addition of steel fibers of different morphologies (SFs and HFs). The principal conclusions from this work are summarized as follows:

(1) UHPS prepared with sand-to-cement ratios of 0.7–0.9, a steel fiber content of 2%, and SSFA replacement ratios ranging from 0% to 75% achieved flowability values of 14–18 cm, which could meet the working performance requirements.
(2) Experiments showed that the sand–cement ratio significantly affected the mechanical properties of UHPS. At the sand–cement ratio of 0.9, the compressive and flexural strengths reached 124.08 MPa and 23.81 MPa, respectively. Replacing MS with steel slag further enhanced the mechanical performance: 75% SSFA increased the compressive and flexural strengths by 1.15 and 1.23 times, respectively, while 50% SSFA achieved the highest splitting tensile strength of 19.57 MPa. Compared with SF, HF improved toughness by 14.2–15% and increased the flexural strength by 20%, the compressive strength by an average of 8.8 MPa, and the splitting tensile strength by an average of 2.5 MPa.
(3) A positive linear correlation was established between the cube compressive strength and uniaxial compressive strength of UHPS incorporating SSFA. At a steel fiber content of 2%, the uniaxial compressive strength was approximately 0.887 times the cube compressive strength. Although this relationship provides a reference for preliminary assessment, further validation using a wider range of mixing ratios is still needed before wider implementation.
(4) The water absorption of the optimal mixture series was investigated. SSFA effectively reduced the water absorption of UHPS. At an SSFA replacement ratio of 75%, the water absorption reached its minimum value, which was 0.5% lower than that of the control group containing only MS.
(5) SEM observations of the aggregate characteristics, ITZ, fiber–matrix interface, and fiber pull-out path provided microstructural evidence for the mechanical performance of UHPS. Compressive strength was predominantly influenced by the SSFA replacement ratio; whereas, steel fiber morphology played a more significant role in improving the splitting tensile strength, flexural strength, and toughness.

The optimized mixture identified in this study exhibits potential for applications requiring high strength and crack resistance, such as tunnel lining and underground support. However, additional durability investigations, including chloride penetration, freeze–thaw resistance, sulfate attack, and long-term performance assessments, are required before its suitability for such engineering applications can be fully validated.

## 5. Limitations and Future Work

Future studies should further investigate chloride penetration resistance, freeze–thaw durability, sulfate resistance, carbonation resistance, long-term shrinkage and water absorption of all experimental groups to comprehensively evaluate the long-term durability of steel slag aggregate UHPS. In addition, quantitative environmental assessments will be conducted in subsequent experiments to validate the sustainability of the research topic.

Although the compressive and flexural strengths continued to improve with increasing SSFA content up to 75%, 100% replacement was not experimentally investigated due to potential workability limitations and experimental condition constraints. Further investigations will be conducted to explore the effects of a 100% replacement of manufactured sand with SSFA on the workability, mechanical performance, and durability of UHPS.

## Figures and Tables

**Table 1:** Chemical composition of cementitious materials (mass %).

Component	Cement	Silica Fume	Fly Ash	Metakaolin
SiO_2_	-	96.6	50.84	54.28
Al_2_O_3_	-	-	33.50	43.83
Na_2_O	-	-	0.30	0.30
K_2_O	-	-	0.90	
MgO	1.01	-	0.51	-
CaO	-	-	2.29	-
Fe_2_O_3_	-	-	3.94	-
Cl^−^	0.018	0.125	-	-

**Table 2:** Main chemical composition of SSFA (mass %).

Component	Fe	CaO	SiO_2_	MgO	Al_2_O_3_	P_2_O_5_	ZrO_2_
content	24.5	48.5	12.1	6.9	4.5	1.55	0.79

**Table 3:** UHPS mix design (kg/m^3^).

Group	Cement	Silica Fume	Fly Ash	Metakaolin	MS	SSFA	W	SF/HF	Superplasticizer
S7G0	840	120	120	120	840	0	240	156	8.4
S7G25	840	120	120	120	630	210	240	156	8.4
S7G50	840	120	120	120	420	420	240	156	9.6
S7G75	840	120	120	120	210	630	240	156	10.8
S8G0	840	120	120	120	960	0	240	156	8.4
S8G25	840	120	120	120	720	240	240	156	8.4
S8G50	840	120	120	120	480	480	240	156	9.6
S8G75	840	120	120	120	240	720	240	156	10.8
S9G0	840	120	120	120	1080	0	240	156	8.4
S9G25	840	120	120	120	810	270	240	156	8.4
S9G50	840	120	120	120	540	540	240	156	9.6
S9G75	840	120	120	120	270	810	240	156	10.8

Note: Except for differences in fiber shape, the other components of different steel fiber groups are completely identical.

**Table 4:** Calculation results of flexural strength performance indicators.

Test Specimen	SF				HF			
	Initial Crack Deflection (mm)	Initial Crack Strength (MPa)	Peak Deflection (mm)	Peak Strength (MPa)	Initial Crack Deflection (mm)	Initial Crack Strength (MPa)	Peak Deflection (mm)	Peak Strength (MPa)
S7G0	0.71	13.79	0.99	15.58	0.71	13.81	0.86	15.83
S7G25	0.72	14.24	1.25	16.20	0.70	14.63	1.04	17.24
S7G50	0.79	14.93	1.16	17.11	0.81	15.05	1.50	19.21
S7G75	0.79	15.45	1.23	18.33	0.82	15.75	1.38	20.00
S8G0	0.77	14.76	1.05	15.67	0.78	14.82	1.46	18.35
S8G25	0.77	14.95	1.13	18.07	0.78	15.64	1.04	19.10
S8G50	0.80	16.60	1.19	18.39	0.80	17.25	1.21	20.69
S8G75	0.90	17.18	1.04	19.01	0.89	18.59	1.43	22.75
S9G0	0.82	15.23	1.22	16.55	0.84	16.60	1.35	19.31
S9G25	0.85	15.05	1.29	18.70	0.85	16.62	1.24	19.85
S9G50	0.90	17.44	1.19	19.23	1.04	17.17	1.73	21.92
S9G75	1.04	17.60	1.52	20.72	1.06	18.24	1.72	23.81

**Table 5:** Calculation results for bending toughness index.

Specimen	SF		HF	
	*I*_5_	*I*_10_	*I*_5_	*I*_10_
S7G0	4.301	5.512	4.333	5.591
S7G25	4.484	5.295	4.682	5.822
S7G50	4.704	5.810	4.812	6.582
S7G75	4.932	6.276	4.982	6.627
S8G0	4.314	5.843	4.663	5.955
S8G25	4.366	5.943	4.703	6.405
S8G50	4.816	6.057	4.717	6.495
S8G75	4.971	6.062	4.751	6.552
S9G0	4.632	5.787	4.722	6.430
S9G25	4.520	5.921	4.919	6.607
S9G50	4.452	5.927	5.084	6.816
S9G75	5.069	6.359	5.269	6.543

## Data Availability

The original contributions presented in the study are included in the article, further inquiries can be directed to the corresponding author.
